# Evaluation of Enzymatically Hydrolyzed *Hermetia illucens* Larval Homogenate as a Dietary Supplement for 1–21-Day-Old Broilers: Growth Performance, Meat Quality, Serum Biochemical Indices and Intestinal Morphology

**DOI:** 10.3390/vetsci13101030

**Published:** 2026-09-29

**Authors:** Zikang Niu, Zujie Liao, Chaohai Yuan, Yafei Cai, Xiaoying Xin, Yujiao Guo, Bingqian Zhou, Niannian Wang, Jiahui Li, Wei Zhang

**Affiliations:** College of Animal Science and Technology, Nanjing Agricultural University, Nanjing 210095, China; 2021205033@njau.edu.cn (Z.N.); 2024105043@stu.njau.edu.cn (Z.L.); 17715597261@163.com (C.Y.); ycai@njau.edu.cn (Y.C.); xinxy1001@163.com (X.X.); yujiaoguo11@163.com (Y.G.); 2024805164@stu.njau.edu.cn (B.Z.); nn12150602@163.com (N.W.); 2025805186@stu.njau.edu.cn (J.L.)

**Keywords:** EH-BSFLH, broiler production performance, serum biochemical indicators, muscle amino acid profile, intestinal morphology

## Abstract

Conventional unprocessed black soldier fly larval meal often exerts inconsistent growth-promoting effects on broilers. This variability may arise from multiple factors, including dietary inclusion level, rearing substrate, processing method, defatting status, and nutritional composition; indigestible macromolecular proteins and chitin are among these contributing factors. Enzymatically hydrolyzed *Hermetia illucens* larval homogenate (EH-BSFLH) is rich in small peptides and lauric acid, which theoretically improve nutrient bioavailability and intestinal health, making it a promising novel functional feed additive. This study aimed to characterize the nutritional profile of a commercially available enzyme hydrolyzed *Hermetia illucens* larval homogenate (EH-BSFLH), and to explore the effects of 2% and 6% dietary EH-BSFLH supplementation on 1–21-day-old Arbor Acres (AA) broilers. A total of 126 one-day-old AA broilers were randomly assigned to three treatments in this 21-day starter feeding trial, with seven pens per treatment. Nutrient composition of EH-BSFLH and multiple phenotypic indicators of broilers were measured and statistically compared. EH-BSFLH utilized in this essay possessed unique nutritional characteristics, including high acid-soluble protein proportion (96.41% relative to total protein), abundant lauric acid (26.62% of total fatty acids), and a calcium-to-phosphorus (Ca/P) ratio of 3.10:1. The 6% EH-BSFLH supplementation increased (*p* < 0.02, FDR < 0.05) 21-d live weight, average daily feed intake (ADFI), average daily gain (ADG), eviscerated yield and semi-eviscerated yield relative to the control, while no significant difference in feed conversion ratio (FCR) was detected across treatments. Dietary EH-BSFLH at 2% and 6% increased (*p* < 0.02, FDR < 0.05) 24 h post-mortem pH of breast and thigh muscles, without affecting meat color or shear force. The 6% EH-BSFLH group elevated (*p* < 0.02, FDR < 0.05) 10 amino acids in thigh muscle, while maintaining stable essential amino acids/total amino acids (EAA/TAA) and essential amino acids/non-essential amino acids (EAA/NEAA) ratios. Moreover, 6% EH-BSFLH decreased duodenal crypt depth and increased Villus Height/Crypt Depth (VH/CD) ratios in duodenum and jejunum (*p* < 0.02, FDR < 0.05). Serum biochemical variables were generally comparable between groups, though differences were observed for glucose, albumin, alanine aminotransferase, aspartate aminotransferase, and total cholesterol (TCHO) (*p* < 0.02, FDR < 0.05). None of these values indicated obvious pathological alterations. This modified insect product exhibits promising application potential for young broiler feeding under the current trial conditions, and the observed shifts in serum metabolic indices may provide partial reference for balancing production performance and broiler physiological status in practical diet formulation.

## Simple Summary

In modern poultry farming, conventional black soldier fly larva feed shows inconsistent effects on broilers, so it is necessary to develop improved insect feed ingredients. This study first analyzed the nutritional components of enzymatically hydrolyzed black soldier fly larval homogenate, which is rich in easily absorbed protein and lauric acid. We then fed one-day-old broilers with diets containing 2% and 6% of this product for 21 days, and evaluated animal growth, meat quality, intestinal health, blood status and economic benefits. The results showed that adding 6% of the product obviously improved broiler growth, carcass traits and meat quality, altered intestinal morphology and yielded a preliminary increase in estimated farming profit under the conditions of this 21-day starter broiler trial. The 2% addition group had no noticeable positive effects. All broilers maintained normal physical conditions throughout the trial. We conclude that the 6% group produced better phenotypic outcomes relative to the control and 2% treatments. This modified insect product has good application prospects in poultry feed, and farmers should balance production performance and animal health during practical use.

## 1. Introduction

Global demand for poultry meat continues to rise, supporting the expansion of intensive broiler production [1]. Feed additives including probiotics, prebiotics, and plant extracts have been applied to improve intestinal health and nutrient assimilation, yet their practical application is constrained by inconsistent efficacy and narrow functional spectra [2,3]. There remains a need to explore novel multifunctional feed ingredients for poultry production [4,5]. Recent studies have further shown that naturally derived dietary supplements can modulate growth performance and selected meat-quality characteristics in poultry, supporting continued investigation of functional feed ingredients as nutritional tools in intensive production systems [6].

Among various natural nutrient sources, insect-derived resources such as *Hermetia illucens* larvae are currently utilized as protein or fat substitute raw materials in animal production [7,8]. *Hermetia illucens*, a dipteran insect in the family Stratiomyidae, is native to tropical and subtropical regions, and now widely distributed in temperate regions around the world [9]. Its larvae have a wide feeding habit, short growth cycle and high biotransformation efficiency, which can efficiently convert organic wastes such as kitchen waste, livestock and poultry manure and agricultural by-products into high-quality insect biomass [10,11]. The dry matter of black soldier fly larvae contains approximately 40–48% crude protein and 25–40% crude fat, and is rich in various bioactive substances including lauric acid, chitin and antimicrobial peptides, which endow them with unique application potential in the feed industry [12,13].

However, the application effects of black soldier fly derived ingredients in broiler production have shown obvious dose-dependent variability, with inconsistent impacts on broiler growth performance, intestinal health and production efficiency in existing studies [14,15,16,17,18]. Importantly, these published results differ substantially depending on ingredient type, including whole larvae, full-fat meal, defatted meal, isolated protein fractions, or enzymatically hydrolyzed products, as these materials differ greatly in nutritional composition and bioactive compound profiles. Low to moderate inclusion levels of *Hermetia illucens* larval meal have been proven to exert positive effects on broiler production: dietary supplementation with appropriate amounts of this meal can improve the starter-phase growth performance of broilers, enhance apparent ileal digestibility of nutrients and optimize intestinal mucosal morphology, which is attributed to its rich nutritional composition and bioactive substances such as lauric acid [18,19,20]. In contrast, excessively high inclusion levels may induce adverse effects on broiler health and production: high-dose addition of *Hermetia illucens* larval meal can reduce the diversity of broiler cecal microbiota, decrease intestinal mucin secretion and even damage intestinal barrier function, thus restricting nutrient absorption and growth performance of broilers [21,22,23]. Additionally, some studies have also found that dietary inclusion of *Hermetia illucens* larval meal within a certain range has no significant effect on the overall growth performance of broilers, though it can slightly regulate the cecal microbial structure and increase breast muscle weight without causing negative impacts on liver function and plasma metabolism [24,25,26].

Such inconsistent application effects are partly related to the incomplete release of bioactive components and the low digestibility of macromolecular substances such as chitin and crude protein in unprocessed *Hermetia illucens* larval meal [5,27,28]. Other important sources of heterogeneity include differences in experimental diets, substitution strategy, broiler age, inclusion level, larval rearing substrate, and processing technology.

Given the inconsistent effects of conventional *Hermetia illucens* larval meal in broiler feeding, EH-BSFLH has been developed as an optimized alternative. Produced from fresh larvae via mild directional enzymatic hydrolysis, this process disrupts chitin structures, releases endogenous proteins and lipids, and degrades macromolecular proteins into small peptides and free amino acids. Notably, this enzymatic treatment also liberates abundant lauric acid, a bioactive component with proven antibacterial activity against intestinal pathogens including *Escherichia coli* and *Salmonella* [29]. Previous poultry trials confirmed that hydrolyzed BSFL can partially substitute dietary fishmeal to boost broiler growth performance and nutrient retention [4]. It should be noted that those studies focused on the fish-meal-replacing potential of protein-enriched hydrolyzed BSFL, whereas the present experimental basal diet contains no fishmeal, and EH-BSFLH is produced via a distinct enzymatic hydrolysis system. The response to alternative feed ingredients can depend markedly on inclusion level, digestive maturity and enzymatic processing, highlighting the importance of evaluating both dose and production phase when interpreting nutritional efficacy [30]. Despite the unique advantages of EH-BSFLH—especially its enriched lauric acid content—systematic research on its application as a functional ingredient in broiler production remains relatively limited, particularly regarding the optimal dietary inclusion level based on its lauric acid content.

In the present study, we first analyzed the basic nutritional profile of a novel product of EH-BSFLH, including conventional nutrients, minerals, amino acids, and fatty acids. The lauric acid proportion of the EH-BSFLH was no less than 26.62% in total fatty acids (TFA). Two inclusion levels (2% and 6%) were selected mainly based on their measured lauric acid concentration. According to the determined lauric acid content of EH-BSFLH, the 2% and 6% dietary inclusion levels theoretically provided approximately 502 mg/kg and 966 mg/kg lauric acid in the complete diet, respectively. Existing literature has confirmed that supplemental lauric acid at 500 and 1000 mg/kg diets could effectively improve broiler intestinal health and growth performance [29]; the 2% and 6% EH-BSFLH dosages were designed to match this verified effective lauric acid dosage range. Subsequently, a feeding trial was conducted on 1–21-day-old AA broilers to evaluate the effects of EH-BSFLH supplementation on growth performance, carcass traits, meat quality, serum biochemical indices, intestinal morphology and economic returns. Given that broiler gut health and feed efficiency are modulated by multiple managerial and nutritional factors [31], findings from this trial may offer experimental evidence for the rational utilization of EH-BSFLH as a functional insect-derived feed ingredient.

## 2. Materials and Methods

All experimental procedures involving live birds were approved by the Institutional Animal Care and Use Committee of Nanjing Agricultural University (Nanjing, China; approved protocol number: SYXK 2025-0096). All chemicals and reagents used were of analytical grade unless otherwise specified.

### 2.1. Preparation of EH-BSFLH

EH-BSFLH (Q/MKSW 003-2025) was manufactured and commercialized by Nantong Mukun Biotechnology Co., Ltd. (Nantong, China). The EH-BSFLH complied with the Chinese Catalogue of Feed Ingredients (Ministry of Agriculture Announcement No. 1773, with subsequent revisions) and the mandatory national hygienic standard for feeds GB 13078-2017 [32]. The corresponding enterprise standard Q/MKSW 003-2025 [33] was developed in accordance with these relevant national feed safety requirements for insect-derived feed ingredients. Routine quality control, including nutritional composition, microbiological indicators and hazardous contaminant testing, is implemented for every production batch to ensure batch-to-batch consistency (Table S1). A single commercial production batch of EH-BSFLH was used throughout the animal trial. In brief, fresh *Hermetia illucens* larvae reared on household and catering food waste were used as raw materials in this study. Both raw larvae and finished EH-BSFLH powder were subjected to microbiological and contaminant testing prior to release. After quality inspection and impurity removal, black soldier fly larvae were rinsed with clean water, blanched in hot water at 90–100 °C for 8–10 min. The blanching water was discarded. Subsequently, the treated larvae were homogenized with fresh water (water dosage equaled 1–2 times the wet weight of larvae) to produce the raw slurry, followed by pH adjustment to 6.0. Enzymatic hydrolysis of the slurry was performed using a proprietary compound enzyme blend consisting of chitinase, trypsin, neutral protease and alkaline protease at a mass ratio of 1:1:3:5. Each individual enzyme component was purchased from commercial suppliers with defined enzyme activity: chitinase, trypsin, neutral protease and alkaline protease (200,000 U/g each). The enzyme mixture was supplemented at 1% of the raw slurry mass. Hydrolysis was carried out at 45–50 °C for 2.5–4 h with continuous stirring, and the pH was maintained at 6.0 during the whole enzymatic reaction. Post-hydrolysis inactivation of the compound enzyme was achieved by heating the hydrolysate to 80 °C and holding the temperature for 30 min. The treated liquid was then sieved through a ≥60-mesh sieve and centrifuged to remove residues. A blend of sodium vitamin C and vitamin E (mass ratio = 1:1, added at 0.2% of filtrate weight) was introduced as an antioxidant into the obtained filtrate, which was subsequently vacuum-concentrated under 0.08 MPa at 50–60 °C to yield black soldier fly slurry with a moisture content of 60%. The concentrated product was further spray-dried to prepare EH-BSFLH powder and preserved under low-temperature storage conditions.

### 2.2. Determination of EH-BSFLH Nutritional Composition

#### 2.2.1. Proximate Nutrients

Moisture: Determined by the constant-weight drying method at 105 °C (GB 5009.3-2016 [34]); *n* = 3.

Crude protein (CP): Determined according to the National Food Safety Standard—Determination of protein in foods (GB 5009.5-2025 [35]) by the Kjeldahl method with a nitrogen-to-protein conversion factor of 6.25; *n* = 3.

Crude fat (CF): Determined according to the National Food Safety Standard—Determination of fat in foods (GB 5009.6-2025 [36]) by Soxhlet extraction with anhydrous diethyl ether; *n* = 3.

Volatile basic nitrogen (VBN): Determined according to the National Food Safety Standard-Determination of total volatile basic nitrogen in food (GB 5009.228-2016 [37]) by the semimicro nitrogen method; expressed as mg/100 g; *n* = 3.

Gross energy (GE): Measured using an automatic oxygen bomb calorimeter (TYHW-V, Hebi Tianyu, Hebi, China); expressed as MJ/kg; *n* = 3.

#### 2.2.2. Fatty Acid Composition Analysis

Total lipids were extracted via the chloroform-methanol method (2:1, *v*/*v*), methylated with 0.5 mol/L KOH-methanol, and analyzed by gas chromatography (GC-2010 Plus, Shimadzu corporation, Kyoto, Japan) using a DB-23 capillary column (30 m × 0.25 mm × 0.25 μm). Nitrogen was used as the carrier gas at a flow rate of 1 mL/min. The temperature program was set as follows: initial temperature of 100 °C held for 5 min, then increased to 200 °C at a rate of 5 °C per minute and held for 10 min, followed by an increase to 230 °C at a rate of 3 °C per minute and held for 10 min. Fatty acids were identified by comparing their retention times with those of a standard mixture (37 FAME Mix, Sigma-Aldrich, Inc., St. Louis, MO, USA) and quantified using the area normalization method in accordance with the National Food Safety Standard—Determination of fatty acids in foods (GB 5009.168-2016 [38]).

#### 2.2.3. Amino Acid Composition

Samples (0.1 g, DM) were hydrolyzed with 6 mol/L HCl at 110 °C for 23 h under nitrogen protection in accordance with the National Food Safety Standard—Determination of amino acids in foods (GB 5009.124-2016 [39]) and AOAC Official Method 994.12. The hydrolysate was filtered, diluted, and analyzed using an amino acid analyzer (LA8080, Hitachi High-Technologies Corporation, Tokyo, Japan) with a lithium ion exchange column. Tryptophan was not determined due to acid hydrolysis degradation; EH-BSFLH: *n* = 4; Thigh muscle: *n* = 5.

#### 2.2.4. Mineral Composition

Samples (0.3–0.5 g) were microwave-digested with 10 mL nitric acid (65%, *v*/*v*) (Mars 6, CEM Corporation, Matthews, NC, USA), and the digest was diluted with 5% nitric acid to a constant volume. Mineral elements (Ca, P, K, Na, Mg, Zn, Cu, Mn) were determined via inductively coupled plasma optical emission spectrometry (ICP-OES 7400, Thermo Fisher Scientific, Inc., Waltham, MA, USA) [40]. The number of replicates was four (*n* = 4).

### 2.3. Animals and Experimental Design

A total of 126 one-day-old male Arbor Acres (AA) broilers with an average initial body weight of 45.17 ± 0.37 g were randomly assigned to 21 pens (6 birds per pen) in a completely randomized design. Birds were divided into three dietary treatments with 7 replicates per treatment: a corn-soybean meal basal diet (control), basal diet supplemented with 2% EH-BSFLH, and basal diet supplemented with 6% EH-BSFLH. No significant difference in initial body weight was detected among groups at the onset of the trial (*p* > 0.05). By analyzing, no significant differences in dietary calcium, total phosphorus, Ca/P ratio, gross energy crude protein (CP) and pH were observed among all groups (*p* = 0.848, *p* = 0.365, *p* = 0.640, *p* = 0.173, *p* = 0.655, and *p* = 0.143). All above relevant data are presented in Table 1 and Table S2. Experimental diets were formulated to meet or approximate the nutritional requirements of broilers as recommended by NRC (1994), with their composition and nutrient levels presented in Table 1.

#### 2.3.1. Animal Housing and Rearing Management

All broilers were raised in three-layer wired cage facilities equipped with automatic temperature and ventilation control systems. Rice husk was used as litter material on the cage bottom mats. Each pen provided usable floor space of 90 × 60 cm, with six birds allocated per pen, maintaining a stocking density of 11 birds per square meter throughout the 21-day trial period. Negative-pressure ventilation was adopted, with continuous low-speed air exchange in the early stage and timed enhanced ventilation after 7 days of age to keep indoor temperature within 28–33 °C and relative humidity at 55–65%.

Daily mortality was recorded. Among the total of 126 experimental broilers, no deaths were observed.

#### 2.3.2. Determination of Dietary Ca/P Ratio

Ca/P ratio was determined following the procedure described in Section 2.2.4 (*n* = 3).

#### 2.3.3. Determination of Gross Energy

GE was determined following the procedure described in Section 2.2.1 (*n* = 3).

#### 2.3.4. Determination of Crude Protein

CP was determined following the procedure described in Section 2.2.1 (*n* = 3).

#### 2.3.5. Determination of pH

Ten grams of samples collected from each group were mixed with 90 mL distilled water, and shaken at 100 r/min for 30 min on a shaker. After filtration, the pH value of the mixture was measured (*n* = 3).

### 2.4. Growth Performance

The experimental period lasted 21 days. All chicks had ad libitum access to feed and water in an environmentally controlled room with a lighting regime of 23 h light and 1 h dark. The room temperature was maintained at 33 °C for the first 7 days, then reduced by 1 °C daily until reaching approximately 28 °C for the remainder of the experiment. At 1 d and 21 d of age, each pen of broilers was weighed as a group. The total live weight of all birds within each replicate pen was divided by the number of birds per pen to calculate average individual body weight (BW) reported in the results. All growth performance variables including Body Weight (BW), Average Daily Feed Intake (ADFI), Average Daily Gain (ADG) and Feed Conversion Ratio (FCR) were analyzed using pen-level data; each individual pen was defined as one independent experimental unit (*n* = 7 per treatment).

ADG = total feed offered − total residual feed (Before weighing leftovers, visible feed spillage and feed refusals on the cage floor were carefully collected and included in residual feed weight to reduce measurement bias)

ADFI = (total feed offered − total residual feed)/(number of birds per pen × days of trial)

FCR = ADFI/ADG

### 2.5. Slaughter Procedure and Measurements

On day 21, one broiler with a BW close to the pen average was selected from each replicate pen (7 independent pen-based biological replicates per treatment; 6 birds were raised in each pen with an initial total of 42 broilers per treatment) and sacrificed by cervical dislocation combined with carotid artery exsanguination (3–4 min). Carcass traits were measured following the method of [41]:

Dressed yield (%) = (Carcass weight/Live BW) × 100 (Carcass weight = Live BW − blood − feathers − claws − beak)

Semi-eviscerated yield (%) = (Semi-eviscerated weight/Live BW) × 100 (Semi-eviscerated weight = Carcass weight − all internal organs)

Eviscerated yield (%) = (Eviscerated weight/Live BW) × 100 (Eviscerated weight = Semi-eviscerated weight + heart + liver + gizzard + abdominal fat)

Breast muscle yield (%) = (Breast muscle weight/Eviscerated weight) × 100

Thigh muscle yield (%) = (Thigh muscle weight/Eviscerated weight) × 100

### 2.6. Meat Quality Assessment

On day 21, one broiler with body weight close to the pen average was selected from each replicate pen, corresponding to 7 independent pen-level biological replicates per treatment (6 birds per pen, 42 broilers in total per treatment) (Table S3). Following slaughter, meat quality parameters of the pectoralis major (breast muscle) and biceps femoris (thigh muscle) were measured:

pH-value: Measured at 24 h post-mortem using a portable pH meter (HI9125, Hanna Instruments, Padua, Italy) with a penetration electrode; three readings were taken per muscle sample (avoiding connective tissue), and the average was calculated [42].

Meat color: Evaluated at 24 h post-mortem using a colorimeter (CR-10 Plus, Konica Minolta, Inc., Tokyo, Japan) with the CIE-Lab system (L* = lightness, a* = redness, b* = yellowness); the instrument was calibrated with a white standard plate before use, and three readings were taken per sample [42].

Shear force: Determined at 96 h post-mortem (4 °C storage) using a digital meat tenderness analyzer (TA.TOUCH, Bosin Technology, Shanghai, China). Muscle samples (1 cm × 1 cm × 3 cm, parallel to muscle fibers) were cooked in a water bath at 80 °C to a core temperature of 70 °C, cooled to room temperature, and sheared perpendicular to muscle fibers; the average of three shear values was recorded (N) [42].

For each group, 5 out of the 7 biological replicates were used for testing. Two replicates were excluded before laboratory analysis following predefined criteria: one maximum outlier and one minimum outlier were removed per group to eliminate extreme values that could distort statistical evaluation. These two replicates were not arbitrarily discarded; the outlier exclusion rule was predefined prior to sample measurement.

### 2.7. Blood Parameter Analysis

#### 2.7.1. Blood Collection

On day 21, all birds were fasted for 12 h overnight before blood collection and slaughter. One broiler with BW close to the pen average was selected from each of the seven replicate pens (6 birds per pen, 42 broilers in total per treatment) as independent biological replicates for wing vein blood collection. All blood collection manipulations were included within the IACUC-approved animal experimental protocol. To alleviate stress and guarantee animal welfare during sampling, trained operators performed venipuncture in a quiet low-light room. Each broiler was softly wrapped with soft fabric for mild restraint, and the whole sampling process was finished within 30 s per bird. Animals were sent back to their original pens immediately after blood collection to avoid sustained stress:

Routine blood samples: 2 mL blood into EDTA anticoagulant tubes (immediately mixed); stored at 4 °C and analyzed within 2 h.

Serum samples: 3 mL blood into non-anticoagulant tubes; allowed to clot at room temperature for 1 h, then centrifuged at 3000× *g* for 15 min at 4 °C; serum was aliquoted and stored at −20 °C until analysis.

#### 2.7.2. Routine Blood Parameters

Analyzed using an automatic hematology analyzer (Rayto RT-7600, Shenzhen, China) for total white blood cell count (WBC), lymphocyte count (LYM), mid-cell count (MID), granulocyte count (GRA), total red blood cell count (RBC), hemoglobin (HGB), and total platelet count (PLT) (*n* = 7).

#### 2.7.3. Serum Biochemical Parameters

Determined using an automatic biochemical analyzer (URIT8000, URIT Medical, Guilin, China) with commercial kits (Nanjing Jiancheng Bioengineering Institute, Nanjing, China) for glucose (GLU), albumin (ALB), alanine aminotransferase (ALT), creatine kinase (CK), triglyceride (TG), aspartate aminotransferase (AST), total bilirubin (T-BIL), uric acid (UA), total protein (TP), and total cholesterol (TCHO) (*n* = 7).

### 2.8. Small Intestinal Morphological Analysis

Preliminary analysis of growth and carcass indices showed that the 2% EH-BSFLH group had no significant differences in most measured traits relative to the control group. Therefore, only the control and 6% EH-BSFLH groups were selected for further intestinal morphological analyses to concentrate detection resources on the treatment with remarkable effects. After slaughter, the small intestine (duodenum, jejunum, ileum) was quickly isolated, and the weight and length of each segment were measured (*n* = 7 per treatment).

Relative weight (g/kg BW) = Intestinal segment weight (g)/Live BW (kg); Relative length (cm/kg BW) = Intestinal segment length (cm)/Live BW (kg)

A 2 cm segment from the middle of each intestinal segment was rinsed with pre-cooled 0.75% physiological saline, fixed in 4% paraformaldehyde for 24 h at 4 °C, and sent to Servicebio Technology Co., Ltd. (Wuhan, China) for paraffin embedding, sectioning (5 μm), and hematoxylin-eosin (HE) staining. Villus height (VH, from villus tip to crypt-villus junction) and crypt depth (CD, from crypt-villus junction to crypt base) were measured under a light microscope (BX53, Olympus Corporation, Tokyo, Japan) with Image-Pro Plus 6.0 software (Media Cybernetics, Rockville, MD, USA). Fifteen intact villi and crypts were measured per sample. These fifteen measurements were averaged to calculate the villus height (VH) and crypt depth (CD) values for each bird, and the VH/CD ratio was subsequently determined prior to statistical analysis.

### 2.9. Economic Benefit Analysis

Economic indicators were calculated to evaluate the economic returns of dietary EH-BSFLH supplementation based on growth performance data and local market prices during the experiment.

Feed cost per kilogram of diet: Calculated according to the inclusion ratio and market unit price of each raw material (basal feed raw materials and EH-BSFLH). These cost parameters are region-specific and tied to local domestic supply chains.

Total feed cost per broiler = Average daily feed intake × trial days × feed cost per kg diet

Live weight revenue per broiler = Final body weight × market selling price of live broilers (CNY/kg)

Net profit per broiler = Live weight revenue per broiler − Total feed cost per broiler

Input-output ratio = Live weight revenue per broiler ÷ Total feed cost per broiler

All market prices used for economic calculation were derived from local commercial broiler feed and livestock markets at the time of this 21-day feeding trial.

### 2.10. Statistical Analysis

All data were tested for normality (Shapiro–Wilk test) and homogeneity of variance (Levene’s test) using SPSS 27.0 software (IBM Corp., Armonk, NY, USA). Residual distributions were visually inspected to further evaluate model assumptions. All datasets in this study met the assumptions of normality and homoscedasticity; thus, one-way analysis of variance (ANOVA) was used for comparisons across the three dietary treatments, and Tukey’s honestly significant difference (HSD) test was used for post hoc multiple comparisons to separate treatment means. Results are presented as mean ± standard deviation (SD). Based on these preliminary growth outcomes, intestinal morphology, serum biochemical parameters, and thigh muscle amino acid composition were subsequently evaluated only between the control and 6% EH-BSFLH groups. Independent-sample *t*-tests were used for these pairwise comparisons. To reduce the risk of Type I error arising from multiple biomarker testing, differences were regarded as statistically significant only when both raw *p* < 0.02 and Benjamini–Hochberg FDR < 0.05.

## 3. Results

### 3.1. Proximate Nutrient Composition and Gross Energy of EH-BSFLH

The proximate nutrient composition and gross energy (GE) of EH-BSFLH are presented in Table 2. EH-BSFLH contained 60.87 ± 0.98% moisture, 15.49 ± 0.71% crude protein (CP), 9.85 ± 0.65% crude fat (CF), and 9.39 ± 0.24 MJ/kg GE. Acid-soluble protein (ASP) constituted 96.41 ± 1.37% of total protein. The VBN content was 96.07 ± 8.78 mg/100 g.

### 3.2. Mineral Element Content of EH-BSFLH

EH-BSFLH was rich in essential macro and trace minerals for broilers (Table 3). Macro mineral contents (mg/kg) were: Na (21,112.40 ± 332.71) > Ca (13,161.54 ± 330.17) > K (5827.84 ± 120.30) > P (4246.98 ± 126.59) > Mg (2198.26 ± 16.82), with a Ca/P ratio of 3.10 ± 0.05. Trace mineral contents (mg/kg) were: Mn (28.64 ± 2.45) > Zn (8.61 ± 1.21) > Cu (1.54 ± 1.05).

### 3.3. Amino Acid Composition of EH-BSFLH

A total of 17 amino acids were detected in EH-BSFLH (Table 4), including 9 EAAs and 8 NEAAs (based on broiler nutritional requirements). TAA content was 29.98 ± 3.38 g/100 g DM, and EAA content was 12.23 ± 1.48 g/100 g DM. The EAA/TAA ratio was 42.20 ± 1.37%, and the EAA/NEAA ratio was 73.09 ± 4.19%. The main EAAs were leucine (2.28 ± 0.29 g/100 g DM) and lysine (2.19 ± 0.24 g/100 g DM), and the main NEAA was glutamic acid (4.77 ± 0.51 g/100 g DM).

### 3.4. Fatty Acid Composition of EH-BSFLH

Ten fatty acids were identified and quantified in EH-BSFLH (Table 5), with a total fatty acid (TFA) content of 223.9 g/kg DM. Saturated fatty acids (SFA) accounted for 123.7 g/kg DM (55.25% of TFA), monounsaturated fatty acids (MUFA) for 59.6 g/kg DM (26.62% of TFA), and polyunsaturated fatty acids (PUFA) for 40.6 g/kg DM (18.13% of TFA). Lauric acid (C12:0) was the most abundant fatty acid at 59.6 g/kg DM (26.62% of TFA), followed by oleic acid (C18:1n9c) at 52.7 g/kg DM (23.54% of TFA) and palmitic acid (C16:0) at 41.1 g/kg DM (18.36 of TFA).

### 3.5. Growth Performance of Broilers

The crude protein and gross energy contents of EH-BSFLH were lower relative to the basal broiler diet. For the purpose of directly evaluating the biological function of EH-BSFLH per se, a top dosing supplementation strategy was adopted rather than re-formulating diets by increasing conventional high-protein or high-energy feed ingredients. This design avoids altering the proportion of other basal components, and the isonitrogenous and isoenergetic status across all final diets was validated by proximate analysis of experimental feed samples. Under this experimental setting, no significant differences in initial BW (1 day) were observed among the three groups (*p* = 0.215, Table 6 and Table S4). At 21 days of age, the 6% EH-BSFLH group had a significantly higher final BW (843.71 ± 39.96 g) than the control (737.14 ± 49.61 g) and 2% group (776.30 ± 38.89 g) (*p* < 0.001, FDR = 0). The 6% group also had significantly higher ADFI (52.47 ± 3.00 g/bird/d) and ADG (38.03 ± 1.91 g/bird/d) than the control (*p* < 0.001, FDR = 0), with no differences between the 2% group and control (*p* = 0.051 and *p* = 0.229). FCR did not differ significantly among the three groups (*p* = 0.631), ranging from 1.37 to 1.40 g/g.

### 3.6. Carcass Traits of Broilers

Dressed yield, breast muscle yield, and thigh muscle yield did not differ significantly among the three groups (*p* > 0.05, Table 7 and Table S5). The 6% EH-BSFLH group had a significantly higher eviscerated yield (79.04 ± 1.67%) and semi-eviscerated yield (88.42 ± 1.34%) than the control (76.10 ± 1.78% and 86.43 ± 1.34%, respectively) (*p* < 0.02) (*p* = 0.015, FDR = 0.0375 and *p* = 0.008, FDR = 0.0375). No significant differences in carcass traits were observed between the 2% group and control (*p* > 0.02).

### 3.7. Meat Quality of Broilers

The 24 h post-mortem pH of breast and thigh muscles was significantly elevated in both EH-BSFLH groups compared with the control (*p* < 0.001, FRD = 0, Table 8 and Table S6). The 6% EH-BSFLH group had a higher breast muscle pH (5.69 ± 0.04) than the 2% EH-BSFLH (5.63 ± 0.02) (*p* = 0.019), with no difference in thigh muscle pH between the two EH-BSFLH groups (*p* = 0.063). No significant differences in meat color (L*, a*, b*) and shear force of breast and thigh muscles were observed among the three groups (*p* > 0.02).

### 3.8. Thigh Muscle Amino Acid Composition of Broilers

Amino acid profiling was performed only on thigh muscle, largely because thigh meat has stronger consumer acceptance and higher market value than breast meat in the Chinese market; relevant results can offer more targeted guidance for local broiler production. Accordingly, thigh muscle was used for amino acid analysis. A two-stage analytical strategy was adopted in this study. First, preliminary analyses of growth performance and carcass indices were conducted for all three dietary treatments (control, 2% EH-BSFLH, and 6% EH-BSFLH). The results showed that the 2% EH-BSFLH group had no significant differences in most measured traits relative to the control group. To concentrate limited detection resources on the treatment with remarkable effects, thigh muscle amino acid composition comparisons were only performed between the control and 6% EH-BSFLH groups. Compared with the control, the 6% EH-BSFLH group had significantly increased contents of 10 amino acids (EAAs and NEAAs) in thigh muscle (*p* < 0.02, FDR < 0.05, Table 9), with no differences in methionine and cysteine (*p* = 0.791 and *p* = 0.210). TAA and EAA contents in the 6% EH-BSFLH group were 14.26 ± 0.37 g/100 g and 6.93 ± 0.20 g/100 g, respectively, representing a 14.9% and 14.7% increase compared with the control (*p* = 0.016, FDR = 0.0344 and *p* = 0.027, FDR = 0.0406). The EAA/TAA (0.49 ± 0.00) and EAA/NEAA (0.95 ± 0.02) ratios did not differ significantly between the two groups (*p* = 0.956 and *p* = 0.949).

### 3.9. Blood Parameters of Broilers

No significant differences in all routine blood parameters (WBC, LYM, MID, GRA, RBC, HGB, PLT, etc.) were observed among the three groups (*p* > 0.05, Table 10 and Table S7). Preliminary analysis of growth and carcass indices showed that the 2% EH-BSFLH group had no significant differences in most measured traits relative to the control group. Therefore, only the control and 6% EH-BSFLH groups were selected for further serum biochemical analyses to concentrate detection resources on the treatment with remarkable effects. However, compared with the control, the 6% EH-BSFLH group had significantly lower serum levels of GLU (11.40 ± 0.43 vs. 12.23 ± 0.37 mmol/L), ALB (11.84 ± 0.63 vs. 13.06 ± 0.36 g/L), ALT (1.90 ± 0.26 vs. 2.62 ± 0.43 U/L), AST (252.30 ± 12.33 vs. 281.06 ± 13.00 U/L), and TCHO (3.49 ± 0.25 vs. 4.07 ± 0.16 mmol/L) (*p* < 0.05, FDR = 0.0320, Table 11). TP, TG and T-BIL levels showed a decreasing trend (*p* > 0.05), and CK, UA, GLB, and A/G ratio did not differ significantly between the two groups (*p* > 0.05).

### 3.10. Intestinal Indices and Morphology of Broilers

The relative weight and relative length of the duodenum, jejunum and ileum of broilers were evaluated to assess intestinal morphological indices (Table 12 and Table S8). No significant differences were observed in duodenal relative weight, ileal relative weight, duodenal relative length, jejunal relative length, and ileal relative length among the three dietary treatments.

**Figure 1:**
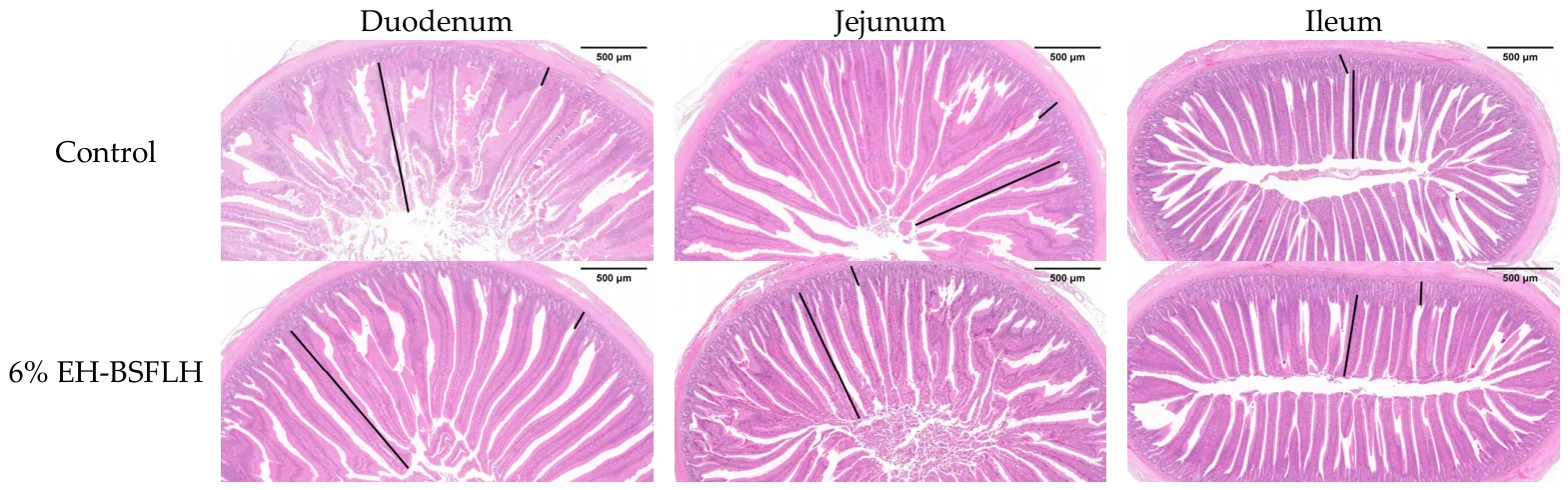
Effects of different supplemental levels of EH-BSFLH on intestinal morphology of broilers. Rows represent dietary treatments: Control (basal diet without EH-BSFLH supplementation) and 6% EH-BSFLH group (basal diet supplemented with 6% EH-BSFLH). Columns correspond to three small intestinal segments: Duodenum, Jejunum and Ileum. In each representative intestinal micrograph, the longer marked line denotes VH, while the shorter marked line indicates CD. Quantitative numerical values of VH and CD for each treatment group are supplemented in the corresponding annotation regions of Figure 1 for intuitive cross-group comparison. All tissue sections were stained with hematoxylin and eosin (H&E). Scale bar = 500 μm. Notably, no significant differences in growth performance were observed between the control group and the 2% EH-BSFLH group; thus, only the control and 6% EH-BSFLH treatments are analyzed and presented here for clearer visual comparison of treatment effects.

To concentrate limited detection resources on the treatment with remarkable effects, intestinal morphological measurements and statistical comparisons were only performed between the control and 6% EH-BSFLH groups. Compared with the control, the 6% EH-BSFLH group had a significantly decreased duodenal CD (177.55 ± 12.85 μm vs. 229.73 ± 22.86 μm) (*p* = 0.012, FDR = 0.0360, Table 13 and Figure 1) and significantly increased VH/CD ratios in the duodenum (10.02 ± 1.10 vs. 6.66 ± 0.94) and jejunum (8.13 ± 0.98 vs. 5.51 ± 1.07) (*p* = 0.004, FDR = 0.0360 and *p* = 0.011, FDR = 0.0360). No significant differences were observed between the two groups in VH of the duodenum and jejunum, and all morphological parameters (VH, CD, VH/CD) of the ileum.

### 3.11. Effects of EH-BSFLH on Economic Returns of Broilers

The effects of EH-BSFLH supplementation on economic returns of broilers are shown in Table 14 and Table S9 and S10. Compared with the control, feed cost, additive cost, total cost, revenue, and profit all increased with higher levels of EH-BSFLH. Notably, the 6% EH-BSFLH group showed significantly higher revenue, and profit than the control and 2% groups (*p* < 0.02, FDR < 0.05), while the 2% group did not differ significantly from the control in most parameters except total cost. The highest profit (CNY 14.05 per broiler) was observed in the 6% EH-BSFLH group. These results indicate that supplementing with 6% EH-BSFLH improved economic returns in broilers. All economic calculations were conducted using the market prices of conventional feed ingredients and EH-BSFLH prevailing within the Chinese feed industry in 2026. It is critical to acknowledge that these cost parameters are region-specific and tied to local domestic supply chains. The economic outcomes and cost-related conclusions presented here are highly sensitive to China’s local market conditions, and such findings may not be generalizable to other nations or regions with divergent feed and insect feedstuff pricing frameworks.

## 4. Discussion

This study characterized the nutritional composition of EH-BSFLH and evaluated its effects when incorporated into diet on 0 to 21-day-old AA broilers. The key findings were that 6% EH-BSFLH supplementation significantly improved broiler growth performance, carcass traits, meat quality, and intestinal health, with a positive economic benefit for commercial production.

### 4.1. EH-BSFLH Nutritional Composition Characteristics

It has been reported that a high content of acid-soluble protein (ASP) can improve the digestion and absorption of animal protein and enhance the growth performance of broilers [43]. In this study, the ASP of EH-BSFLH accounted for 96.41% of the total protein, which may indicate protein degradation yielding soluble peptide-rich fractions. The VBN content (96.07 ± 8.78 mg/100 g) was below the limit specified for high-grade fish meal in the Chinese National Standard (GB/T 19164-2021 [44]) and within the acceptable range reported for fish meal quality in international guidelines (typically <150 mg/100 g) [45]. These compositional characteristics support the potential suitability of EH-BSFLH as a source for animal feed applications. Lauric acid, which has been documented to exert beneficial effects on intestinal function and nutrient utilization in broilers, accounted for 26.62% of the total fatty acids (TFA) in EH-BSFLH [46]. In addition, EH-BSFLH is rich in various mineral elements critical for bone development and physiological metabolism in broilers, and given its inherent Ca/P ratio of 3.10:1, the dietary Ca/P ratios determined in the 2% and 6% treatment groups (1.54:1 and 1.52:1, respectively) fell within the optimal range of 1.5:1 to 2.0:1 recommended for AA broilers from 1 to 21 days of age to ensure efficient mineral absorption [27,47,48].

Nevertheless, EH-BSFLH in this study had a CP content of only 15.49% and GE of 9.39 MJ/kg, which were markedly lower than the CP (~22.61%) and GE (~18.09 MJ/kg) of the basal broiler diet. For this reason, a top-dosing supplementation strategy was adopted in this study, rather than elevating other high-protein and high-energy feed ingredients to achieve theoretical isonitrogenous and isoenergetic diets, as the former approach ensures that any differences in broiler growth performance could be attributed exclusively to EH-BSFLH. Although EH-BSFLH addition brings extra nutrients into the diet, on the other hand, its overall contribution to the total nutrient supply of the complete mixed feeds remained limited. Combined with unavoidable minor weighing errors during large-scale feed mixing, proximate analysis of the experimental diets revealed no statistically significant differences in crude protein and gross energy among treatment groups.

It should nevertheless be acknowledged that external top-supplementation cannot completely eliminate small nutrient deviations across diets, which constitutes a potential confounding factor for interpreting nutritional trial outcomes. Even though chemical testing of stored feed samples demonstrated that such nutrient discrepancies were minor in our experiment, the top-dosing design cannot achieve theoretically strict isonitrogenous and isoenergetic conditions. We have clearly noted this limitation and suggest that future studies adopt ingredient-replacement schemes to produce strictly isonitrogenous and isoenergetic diets when evaluating the nutritional efficacy of insect-derived feed additives.

### 4.2. Effects of EH-BSFLH on Broiler Growth Performance

Growth performance is the most intuitive indicator for evaluating the application effect of feed supplements, wherein ADG, ADFI, and FCR directly reflect breeding efficiency. In the present study, broilers in the 6% EH-BSFLH group exhibited significantly greater 21-day BW, ADFI, and ADG compared with the control group, whereas the 2% EH-BSFLH group showed no significant promoting effects, indicating that there is an effective dosage threshold for functional component supplementation. No significant difference in FCR was observed among all groups, which was mainly attributed to the synchronous increase in feed intake and weight gain. These results indicate that 6% EH-BSFLH increased broiler growth at least by stimulating feed intake. One plausible explanation is that EH-BSFLH may alter diet palatability and consequently raise voluntary feed intake, and the elevated final body weight largely arises from greater total nutrient consumption, instead of enhanced nutrient utilization efficiency. Recent broiler studies using enzyme-supported alternative feed ingredients have shown that direct measurements of crude protein digestibility and metabolizable energy are valuable for distinguishing improvements in nutrient utilization from changes driven primarily by feed intake or dietary inclusion level [30]. The results indicate that the 6% supplementation level was effective under the conditions of this study, but they do not allow the determination of an optimal or threshold dose. Previous broiler trials using BSFL-derived products (including hydrolyzed BSFL materials) have examined higher dietary inclusion rates ranging from 10% to 20%, and reported variable outcomes: high inclusion of BSFL products may yield limited benefits or even compromise growth performance [49]. Further dose–response studies with wider gradients, including 10–20% EH-BSFLH, are required to identify the optimal dosage.

Notably, Dabbou et al. (2018) reported improved growth performance and altered intestinal histological structure in broilers fed conventional defatted black soldier fly larval meal [40]. Compared with the whole defatted insect meal used in their trial, the enzymatically hydrolyzed EH-BSFLH in the present study contains abundant small peptides and free fatty acids, which may exert more potent beneficial effects on broiler production and intestinal function, highlighting the advantages of enzymatic hydrolysis processing for insect feed ingredients.

### 4.3. Effects of EH-BSFLH on Broiler Carcass Traits

Carcass traits are important economic characteristics in meat-type animal production and eviscerated yield and semi-eviscerated yield directly determine carcass output [50]. In the present study, 6% EH-BSFLH significantly increased eviscerated yield and semi-eviscerated yield of broilers, but had no significant effects on dressed yield, breast muscle yield, or thigh muscle yield. We hypothesize that these differences may be related to optimized intestinal morphology and improved nutrient absorption, which could potentially modify body tissue deposition efficiency [51]. These findings are also consistent with previous studies reporting that black soldier fly larval meal improves carcass traits in broilers [52].

### 4.4. Effects of EH-BSFLH on Broiler Meat Quality

The 2% and 6% EH-BSFLH groups both showed significantly increased pH values in breast and thigh muscles at 24 h post-mortem. Such pH changes may bring potential benefits to meat quality, whereas no remarkable variations were found in other key meat quality indicators including color and shear force in this trial. Muscle pH post-mortem is a key indicator reflecting meat freshness, water-holding capacity, and tenderness [53]. A higher muscle pH inhibits anaerobic glycolysis of muscle glycogen, reduces lactic acid accumulation, delays meat spoilage, and reduces drip loss [54]. This elevation in muscle pH may be associated with bioactive compounds in EH-BSFLH, such as lauric acid and small peptides, which have been reported to regulate muscle cell energy metabolism and improve the redox status of broilers [55]. No significant differences in meat color and shear force were observed among groups, implying that EH-BSFLH barely altered these major sensory properties of broiler meat. Dietary functional ingredients may influence individual meat-quality parameters differently and improvements in one parameter do not necessarily imply a generalized enhancement of meat quality as responses can vary across pH, oxidative stability, color, texture and sensory characteristics [6]. Whether dietary EH-BSFLH can enhance meat freshness remains to be verified via dedicated freshness biomarkers, and the meat performance of all treatments can meet conventional commercial broiler production standards.

### 4.5. Effects of EH-BSFLH on Broiler Muscle Amino Acid Composition

Muscle amino acid profile is a core indicator for evaluating meat nutritional value, and its content and composition directly affect meat nutritional quality and flavor [56]. In the present study, EH-BSFLH showed a comprehensive amino acid profile, balanced essential amino acids and high contents of small peptides. These intrinsic characteristics might be conducive to intestinal amino acid utilization and provide extra substrates for muscle amino acid deposition, while the enhancement of absorption efficiency cannot be confirmed without dedicated detection in our trial.

Regarding individual amino acid changes, the 6% EH-BSFLH group showed a significant increase in thigh muscle glutamic acid, consistent with the high glutamic acid content in EH-BSFLH raw material; similarly, histidine, alanine, leucine, lysine and other amino acids were also markedly elevated, matching EH-BSFLH’s abundance in these amino acids. The 6% EH-BSFLH significantly increased 10 out of 17 amino acids in thigh muscle. Methionine, threonine, alanine, cysteine, glycine, proline, and tyrosine were not significantly affected, while tryptophan was not determined in this assay, as it was degraded under acid hydrolysis conditions. Total amino acids (TAA) and essential amino acids (EAA) increased by 14.9% and 14.7%, respectively, while EAA/TAA and EAA/NEAA ratios remained stable. These results indicated that EH-BSFLH directly enhanced muscle amino acid deposition via its high amino acid content and maintained the body’s inherent amino acid balance without disturbing amino acid homeostasis, consistent with previous findings that black soldier fly supplementation increases muscle amino acid deposition while preserving the normal muscle amino acid profile, thereby improving meat nutritional quality and flavor [57,58].

### 4.6. Effects of EH-BSFLH on Broiler Blood Parameters

Blood parameters can objectively reflect animal health status, liver function, lipid metabolism, and nutritional status [59]. The present study found that all hematological parameters in each supplemented group were within physiological ranges similar to the control, indicating that EH-BSFLH had no adverse effects on immune function and hematopoiesis.

Significantly lower serum ALT and AST were detected in the 6% treatment group, which may suggest potential shifts in hepatic metabolism. Nevertheless, ALT exhibits limited diagnostic specificity in poultry, and AST is not liver-specific. Hence, these variations alone cannot conclusively demonstrate reduced hepatocellular damage, and such declines are tentatively hypothesized to be associated with antioxidant components (lauric acid) contained in EH-BSFLH. Based on previous reports, lauric acid could scavenge reactive oxygen species and relieve hepatic oxidative stress, whereas no oxidative stress or antioxidant indicators were measured in this study to verify this speculation [60]. By comparing our serum lipid results of 2% and 6% EH-BSFLH supplementation with previous studies, we tentatively propose a potential hormetic response of medium-chain fatty acids: low-to-moderate lauric acid concentrations corresponding to 2% EH-BSFLH maintained stable lipid homeostasis with no notable differences from the control group, while higher lauric acid levels in the 6% EH-BSFLH group may exert stronger lipid-regulating effects through multiple molecular pathways reported in other research [29,61]. From published mechanistic studies, lauric acid may act as a PPARα ligand to upregulate fatty acid oxidation and ketogenesis-related genes [62]. It may also elevate AMPK activity to restrain de novo lipogenesis, suppress SREBP-1c expression to reduce hepatic triglyceride synthesis, and modulate APOB expression to alter VLDL assembly and secretion in a dose-dependent fashion [63]. In addition, prior gut-liver axis research indicated that lauric acid could reshape intestinal microbiota (increasing *Lactobacillus* and decreasing *Escherichia-Shigella*) and indirectly modulate hepatic lipid metabolism by adjusting SCFA production and mitigating endotoxin release [62]. However, all above molecular pathways remain to be confirmed in our experimental trial. The significant decrease in serum TCHO and numerical declining trend of TG indicated a mild lipid-lowering tendency of EH-BSFLH supplementation. This phenomenon may be partially linked to unsaturated fatty acids (linoleic acid) contained in *Hermetia illucens* larvae. Published broiler trials have reported variable responses to linoleic acid supplementation: many studies demonstrated that linoleic acid can reduce serum total cholesterol and triglyceride levels by inhibiting hepatic lipid synthesis, promoting cholesterol excretion, and accelerating fatty acid oxidation, although inconsistent outcomes have also been documented [64,65]. The slight decrease in serum ALB remained within the physiological normal range, and no intergroup differences were detected in TP and GLB, indicating no risk of nutritional deficiency across all treatments [66].

The overall decreasing trend of these indices reflects a multi-dimensional dynamic metabolic response in broilers fed EH-BSFLH. Dietary EH-BSFLH increased broiler growth performance and altered several serum biochemical indices related to nutrient metabolism (GLU, ALB, ALT, AST, TCHO). These serum changes should be interpreted cautiously and do not necessarily represent beneficial metabolic modifications. These findings may offer a preliminary perspective on balancing production performance and animal physiological status when selecting EH-BSFLH supplementation levels.

### 4.7. Effects of EH-BSFLH on Broiler Intestinal Morphology

Intestinal health is the foundation of broiler growth and development, and the VH/CD ratio is a key morphological marker reflecting intestinal structural integrity. The 6% EH-BSFLH supplementation significantly decreased duodenal CD and increased VH/CD ratios in the duodenum and jejunum, reflecting improved intestinal mucosal architecture. Notably, these morphological changes alone cannot directly confirm enhanced nutrient absorption, intestinal barrier integrity, or overall intestinal health; such functional conclusions require additional complementary functional measurements. A decreased CD indicates reduced intestinal epithelial cell proliferation and maintenance costs, while an increased VH/CD ratio indicates an expanded nutrient absorption area and improved epithelial cell renewal efficiency [67]. This beneficial effect may be attributed to the combined effects of lauric acid and small peptides in EH-BSFLH: lauric acid enhances the intestinal mucosal barrier and reduces inflammatory responses, while small peptides promote the proliferation and differentiation of intestinal epithelial cells and repair the intestinal mucosal structure [29,68]. No significant changes in ileal morphology were observed, which may be due to the different physiological functions of intestinal segments (the duodenum and jejunum are the main sites of nutrient absorption, while the ileum is the main site of nutrient reabsorption and microbial colonization). These results are consistent with previous findings that moderate dietary supplementation with black soldier fly larva meal promotes intestinal health in broilers [69,70]. However, one limitation of this trial is that we failed to measure cecal microbiota, so we cannot further clarify the mediating role of intestinal microorganisms in the intestinal protective effects of EH-BSFLH. Another statistical limitation arises from this two-stage, data-dependent analytical strategy. Intestinal morphology was only compared between the control and 6% EH-BSFLH groups after preliminary evaluation of growth and carcass data across all three groups. Although no studies are currently available on these aspects for chicken feeds integrated with EH-BSFLH, this exploratory post hoc selection of comparison groups may inflate the family-wise Type I error rate and increase the probability of false-positive findings. To mitigate the risk of Type I error caused by multiple biomarker testing, we adopted a dual threshold criterion: differences were considered statistically significant only when both raw *p* < 0.02 and Benjamini–Hochberg FDR < 0.05.

### 4.8. Effects of EH-BSFLH on Economic Returns of Broilers

This study demonstrated that dietary supplementation with EH-BSFLH, particularly at the 6% level, increased both total revenue and profit per bird compared with the control group, despite the associated increases in feed cost and additive cost. These findings suggest that, under this specific market scenario, EH-BSFLH can serve as a cost-effective feed ingredient that improves the overall economic returns of broiler production. A sensitivity analysis was not performed in the current study, and the profit outcome may change substantially if the price of EH-BSFLH or live broilers fluctuates.

Similar results have been reported in the literature. Mutisya et al. found that replacing fish meal with black soldier fly larvae meal (BSFLM) in broiler diets increased net profit per bird proportionally to the inclusion level, leading to higher farmer profits [71]. Ahmed et al. observed that supplementing *Hermetia illucens* larvae at 8% and 12% improved the cost–benefit ratio and return on investment in ROSS-308 broilers while also reducing cecal *E. coli* and *Salmonella* counts [53]. In the present study, although the 6% EH-BSFLH group incurred higher total costs than the control group (15%), the increases in revenue (14%) and profit indicate (14%) that the additional income outweighed the extra expenses. This economic trend is consistent with previous research by Waithaka et al., who reported a cost–benefit ratio of 2.12 in improved indigenous chickens fed diets supplemented with 20% conventional BSFL meal as fishmeal replacement. Notably, our trial achieved a higher cost–benefit ratio of 2.99 in commercial broilers with only 6% dietary EH-BSFLH, demonstrating promising economic performance economic efficiency at a much lower inclusion level compared with the conventional BSFL product.

The economic feasibility of BSFL inclusion, however, is influenced by multiple factors, including local prices of conventional feed ingredients, the cost of insect meal production, and the production system [72]. The economic analysis in our trial was calculated based on local market prices during the experimental period, and this economic outcome is scenario-specific and cannot be generalized to other regions or different market periods. Profit estimates are highly sensitive to fluctuations in EH-BSFLH and live broiler prices. In the present study, 6% dietary EH-BSFLH supplementation proved profitable; nevertheless, producers should adjust the inclusion level according to local feed costs and rearing conditions to optimize profitability.

### 4.9. Limitations of the Present Study

Several key limitations of this trial should be acknowledged to frame the scope and generalizability of our findings:

First, the feeding trial only covered a short 21-day starter period, and the effects of EH-BSFLH on grower-finisher performance (21–42 days) and overall flock performance remain unknown. Extended full-cycle feeding trials are required to validate the sustained benefits of this additive.

Second, all experimental animals were limited to male Arbor Acres broilers. The single breed and single sex of broilers restrict the extrapolation of our conclusions to female chickens, other commercial broiler strains, or dual-purpose poultry varieties.

Third, we adopted a top-supplementation strategy rather than fully reformulating the diets. This design inevitably changes the relative proportion of basal feed ingredients after normalization, which should be considered when interpreting treatment responses. We did not conduct apparent total tract nutrient digestibility measurements in this study. Without direct digestibility data, all inferences linking improved intestinal morphology to enhanced nutrient absorption remain indirect and unconfirmed.

Fourth, intestinal microbiota sequencing and profiling were not performed, and intestinal barrier function markers were not quantified. We cannot identify whether EH-BSFLH reshapes gut microbial composition or modulates intestinal barrier integrity to mediate the observed intestinal and growth benefits, which restricts mechanistic interpretation of intestinal responses.

Fifth, the biomarker panel in this trial was incomplete for systemic stress and inflammation assessment. Although hepatic antioxidant indicators were detected, circulating inflammatory cytokines and systemic oxidative stress markers were not measured, so we cannot fully elucidate the systemic anti-stress or anti-inflammatory effects of EH-BSFLH supplementation.

Sixth, we analyzed meat pH as an indicator related to meat freshness, yet no meat shelf-life storage tests were carried out. Long-term chilled storage evaluation of meat quality is needed to further confirm whether the modified muscle pH observed in the 6% EH-BSFLH group translates to prolonged shelf life.

Seventh, a greater number of replicates and more broilers per cage could further improve statistical robustness. In the present trial the number of broilers was restricted by practical breeding conditions and animal welfare regulations, which otherwise may cause overcrowding, triggering chronic stress, aggravated intestinal injury and intense feeding competition. Accordingly, we suggest that future relevant feeding trials adopt a new experimental regulation with additional replicate pens and more birds per unit, enabling more sensitive identification of mild or medium phenotypic differences between dietary treatments.

Eighth, intestinal morphology, serum biochemistry, and amino acid analyses were restricted to the control and 6% EH-BSFLH groups instead of all three treatments. Subgroup selection for these biomarker measurements was undertaken after obtaining preliminary growth data, rendering these pairwise comparisons exploratory post hoc analyses.

Collectively, these limitations create boundaries for the mechanistic and applied conclusions drawn herein, and they provide clear directions for future follow-up research on EH-BSFLH as a poultry feed additive.

## 5. Conclusions

EH-BSFLH utilized in this study is an insect-derived ingredient abundant in acid-soluble protein, lauric acid, balanced amino acid profiles and essential minerals. Compared with the control and 2% groups, dietary supplementation with 6% EH-BSFLH in 0 to 21-day-old AA broilers increased feed intake and body weight gain and altered selected carcass traits, 24 h post-mortem muscle pH, thigh muscle amino acid profiles, and intestinal morphological indices, including decreased duodenal crypt depth and elevated duodenal and jejunal VH/CD ratios. Of note, FCR was comparable across all treatments, suggesting that the observed improvement in growth performance was associated with increased feed intake during the 1–21-d starter period. Within the two tested inclusion levels, 6% EH-BSFLH yielded relatively favorable broiler performance and generated partial economic returns under our experimental conditions. Nevertheless, the economic benefits and commercial application potential observed in the present study should be regarded as preliminary. Given the alterations in serum biochemical profiles, a balance between growth promotion and physiological status should be considered when using EH-BSFLH in broiler starter diets. Future studies are warranted to verify long-term effects across the full production cycle, screen precise optimal dosage via multi-gradient dose–response design, and elucidate the underlying molecular mechanisms.

## Figures and Tables

**Table 1:** Experimental diet formula and nutrient level.

Items	Dietary Treatments ^(3)^			*p*-Value
	Control	2% EH-BSFLH	6% EH-BSFLH	
Ingredients, %				
EH-BSFLH	0.00	2.00 (1.96)	6.00 (5.66)	
Corn	57.20	57.20 (56.08)	57.20 (53.96)	
Soybean meal	31.81	31.81 (31.19)	31.81 (30.01)	
Corn gluten meal	3.40	3.40 (3.33)	3.40 (3.21)	
Soybean oil	3.10	3.10 (3.04)	3.10 (2.92)	
Dicalcium phosphate	1.40	1.40 (1.37)	1.40 (1.32)	
Limestone	1.35	1.35 (1.32)	1.35 (1.27)	
Salt	0.30	0.30 (0.29)	0.30 (0.28)	
L-Lysine	0.25	0.25 (0.25)	0.25 (0.24)	
DL-Methionine	0.19	0.19 (0.19)	0.19 (0.18)	
Premix ^(1)^	1.00	1.00 (0.98)	1.00 (0.94)	
Total	100	102 (100)	106 (100)	
Calculated nutrient levels				
Metabolizable energy, MJ/kg	12.50	——	——	
Gross Energy, MJ/kg	18.09	18.27 (17.91)	18.65 (17.59)	
Crude protein, %	22.61	22.73 (22.28)	22.97 (21.67)	
Calcium, %	1.00	1.01 (0.99)	1.03 (0.97)	
Zinc, mg/kg	75	75.06 (73.59)	75.20 (70.94)	
Total phosphorus, %	0.71	0.72 (0.71)	0.73 (0.69)	
Lysine, %	1.17	1.19 (1.17)	1.22 (1.15)	
Methionine, %	0.50	0.50 (0.49)	0.51 (0.48)	
Methionine + cysteine, %	0.87	0.87 (0.85)	0.90 (0.85)	
Analytical nutrient levels ^(2)^				
Calcium, mg/kg	9231.90 ± 139.42	9285.00 ± 206.12	9302.19 ± 160.70	0.848
Total phosphorus, mg/kg	6001.40 ± 105.52	6030.30 ± 72.40	6105.33 ± 72.21	0.365
Ca/P	1.54 ± 0.03	1.54 ± 0.03	1.52 ± 0.01	0.640
Gross Energy, MJ/kg	18.16 ± 0.11	18.42 ± 0.43	18.70 ± 0.30	0.173
Crude protein, %	22.42 ± 0.39	22.60 ± 0.50	22.74 ± 0.31	0.655
pH	5.80 ± 0.02	5.79 ± 0.01	5.77 ± 0.01	0.143

Note: ^(1)^ Provided per kilogram of diet: Vitamin A, 10,000 IU; Vitamin D, 3000 IU; Vitamin E, 30 IU; Vitamin K3, 1.5 mg; Vitamin B1, 2.1 mg; Vitamin B2, 9 mg; Vitamin B3, 35 mg; Calcium pantothenate, 12 mg; Vitamin B6, 3 mg; Biotin, 0.1 mg; Folic acid, 1.2 mg; Vitamin B12, 0.02 mg; Iron (Fe), 80 mg; Copper (Cu), 10 mg; Manganese (Mn), 85 mg; Zinc (Zn), 75 mg; Iodine (I), 1.15 mg; Selenium (Se), 0.3 mg; ^(2)^ Nutritional levels were analyzed and confirmed on day 0, 14 and 21 of the animal experiment. ^(3)^ All basal dietary ingredients summed to 100% and remained identical across all treatments. EH-BSFLH was top-supplemented at inclusion rates of 2% and 6% based on the mass of the basal diets, resulting in total ingredient sums of 102% and 106% for the two supplemented groups, respectively. Values in parentheses denote ingredient proportions and nutrient levels normalized to a uniform 100% complete mixed diet basis (original values divided by 1.02 for the 2% EH-BSFLH group and divided by 1.06 for the 6% EH-BSFLH group). Data are presented as mean ± standard deviation (SD) (*n* = 3).

**Table 2:** Proximate nutritional composition of EH-BSFLH.

Parameters ^(1)^	EH-BSFLH ^(2)^
M, %	60.87 ± 0.98
CP, %	15.49 ± 0.71
CF, %	9.85 ± 0.65
ASP/TP, %	96.41 ± 1.37
VBN, mg/100 g	96.07 ± 8.78
GE, MJ/Kg	9.39 ± 0.24

Notes: ^(1)^ M, moisture; CP, crude protein; CF, crude fat; ASP/TP, acid-soluble protein/total protein; VBN, volatile basic nitrogen; GE, gross energy. ^(2)^ EH-BSFLH: Enzymatically Hydrolyzed *Hermetia illucens* Larval Homogenate. Data are expressed as mean ± standard deviation (*n* = 3).

**Table 3:** Mineral element contents of EH-BSFLH (mg/kg).

Minerals ^(1)^	EH-BSFLH
K	5827.84 ± 120.30
Na	21,112.40 ± 332.71
Zn	8.61 ± 1.21
Cu	1.54 ± 1.05
Mn	28.64 ± 2.45
Mg	2198.26 ± 16.82
Ca	13,161.54 ± 330.17
P	4246.98 ± 126.59
Ca/P	3.10 ± 0.05

Notes: ^(1)^ K, potassium; Na, sodium; Zn, zinc; Cu, copper; Mn, manganese; Mg, magnesium; Ca, calcium; P, phosphorus. Data are expressed as mean ± standard deviation (*n* = 4).

**Table 4:** Amino acid contents of EH-BSFLH (g/100 g DM).

Amino Acids ^(1)^	EH-BSFLH
EAA	
Arginine	0.65 ± 0.32
Histidine	0.70 ± 0.19
Isoleucine	1.37 ± 0.14
Leucine	2.28 ± 0.29
Lysine	2.19 ± 0.24
Methionine	0.35 ± 0.23
Phenylalanine	1.31 ± 0.11
Threonine	1.27 ± 0.11
Valine	2.12 ± 0.30
NEAA	
Alanine	2.60 ± 0.34
Aspartic Acid	2.87 ± 0.38
Cysteine	0.48 ± 0.16
Glutamic Acid	4.77 ± 0.51
Glycine	1.12 ± 0.19
Proline	1.04 ± 0.17
Serine	1.25 ± 0.17
Tyrosine	1.02 ± 0.13
TAA	29.98 ± 3.38
EAA	12.23 ± 1.48
EAA/TAA, %	42.20 ± 1.37
EAA/NEAA, %	73.09 ± 4.19

Notes: ^(1)^ EAA, essential amino acids; NEAA, non-essential amino acids; TAA, total amino acids. Data are expressed as mean ± standard deviation (*n* = 4).

**Table 5:** Fatty acid contents of EH-BSFLH (g/kg DM) ^(1)^.

Fatty Acids ^(2)^	EH-BSFLH
Capric acid C10:0	3.1
Lauric acid C12:0	59.6
Myristic acid C14:0	11.4
Palmitic acid C16:0	41.1
Palmitoleic acid C16:1	6.9
Stearic acid C18:0	8.5
Oleic acid C18:1n9c	52.7
Linoleic acid C18:2n6c	35.7
α-Linolenic acid C18:3n3	3.5
Arachidonic acid C20:4 n6c	1.4
SFA	123.7
MUFA	59.6
PUFA	40.6
TFA	223.9
Lauric acid/TFA, %	26.62

Notes: ^(1)^ Fatty acid profiling was analyzed using a composite sample pooled from three independent production batches of EH-BSFLH. ^(2)^ SFA, saturated fatty acids; MUFA, monounsaturated fatty acids1; PUFA, polyunsaturated fatty acids; TFA, total fatty acids.

**Table 6:** Effects of supplemental levels of enzymatically EH-BSFLH on growth performance of broilers.

Parameters ^(1)^	Dietary Treatments ^(2)^			*p*-Value	FDR
	Control	2% EH-BSFLH	6% EH-BSFLH		
1d LW, g	45.32 ± 0.45	45.19 ± 0.39	44.99 ± 0.21	0.215	0.2687
21d LW, g	737.14 ± 49.61 ^a^	776.30 ± 38.89 ^a^	843.71 ± 39.96 ^b^	<0.001	0
ADFI, g	45.10 ± 2.66 ^a^	48.68 ± 2.18 ^a^	52.47 ± 3.00 ^b^	<0.001	0
ADG, g	32.94 ± 2.37 ^a^	34.81 ± 1.84 ^a^	38.03 ± 1.91 ^b^	<0.001	0
FCR, g/g	1.37 ± 0.07	1.40 ± 0.05	1.38 ± 0.03	0.631	0.6310

Notes: ^(1)^ LW, live weight; ADFI, average daily feed intake; ADG, average daily gain; FCR, feed conversion ratio. ^(2)^ 2% EH-BSFLH and 6% EH-BSFLH represent the experimental diets supplemented with 2% and 6% EH-BSFLH (1–21 days), respectively. Values with different superscript letters in the same row differ significantly (*p* < 0.02 and FDR < 0.05). Data are expressed as mean ± standard deviation. Each replicate was regarded as an experimental unit for statistical analysis (*n* = 7).

**Table 7:** Effects of different supplemental levels of EH-BSFLH on slaughter performance of broilers.

Parameters	Dietary Treatments			*p*-Value	FDR
	Control	2% EH-BSFLH	6% EH-BSFLH		
Carcass yield, %	91.75 ± 1.21	92.96 ± 2.26	93.30 ± 0.96	0.180	0.3000
Eviscerated yield, %	76.10 ± 1.78 ^a^	77.01 ± 1.69 ^ab^	79.04 ± 1.67 ^b^	0.015	0.0375
Semi-eviscerated yield, %	86.43 ± 1.34 ^a^	86.07 ± 1.40 ^a^	88.42 ± 1.34 ^b^	0.008	0.0375
Breast muscle yield, %	21.95 ± 2.55	21.93 ± 2.01	22.17 ± 0.76	0.845	0.9680
Thigh muscle yield, %	17.86 ± 1.69	18.29 ± 1.28	18.10 ± 1.08	0.968	0.9680

Notes: Values with different superscript letters in the same row differ significantly (*p* < 0.02 and FDR < 0.05). Data are expressed as mean ± standard deviation. Each replicate was used as an experimental unit for statistical analysis (*n* = 7).

**Table 8:** Effects of different supplemental levels of EH-BSFLH on meat quality of broilers.

Parameters	Dietary Treatments			*p*-Value	FDR
	Control	2% EH-BSFLH	6% EH-BSFLH		
pH, (24 h)					
Breast muscle	5.56 ± 0.04 ^a^	5.63 ± 0.02 ^b^	5.69 ± 0.04 ^c^	<0.001	0
Thigh muscle	5.65 ± 0.05 ^a^	5.72 ± 0.01 ^b^	5.77 ± 0.04 ^b^	<0.001	0
Redness, (a*)					
Breast muscle	9.28 ± 1.36	9.90 ± 1.18	10.73 ± 1.61	0.179	0.5307
Thigh muscle	10.58 ± 1.29	11.75 ± 1.51	12.16 ± 1.70	0.157	0.5307
Yellowness, (b*)					
Breast muscle	12.13 ± 1.55	12.44 ± 1.92	13.09 ± 1.54	0.558	0.5580
Thigh muscle	13.62 ± 1.77	14.50 ± 1.57	14.88 ± 1.87	0.398	0.5307
Lightness, (L*)					
Breast muscle	49.41 ± 1.94	48.29 ± 2.81	47.86 ± 0.91	0.336	0.5307
Thigh muscle	50.77 ± 2.79	49.77 ± 1.84	48.99 ± 1.63	0.321	0.5307
Shear force, (N)					
Breast muscle	25.45 ± 1.83	25.77 ± 1.47	24.30 ± 2.23	0.326	0.5307
Thigh muscle	23.00 ± 1.15	23.97 ± 1.98	22.76 ± 2.87	0.538	0.5580

Notes: Values with different superscript letters in the same row differ significantly (*p* < 0.02 and FDR < 0.05). Data are expressed as mean ± standard deviation. Each replicate was regarded as an experimental unit for statistical analysis (*n* = 7).

**Table 9:** Effects of different supplemental levels of EH-BSFLH on muscle amino acid composition of broilers.

Amino Acids ^(1)^	Dietary Treatments		*p*-Value	FDR
	Control	6% EH-BSFLH		
EAA				
Arginine	0.83 ± 0.07 ^a^	0.96 ± 0.03 ^b^	0.010	0.0315
Histidine	0.40 ± 0.05 ^a^	0.51 ± 0.05 ^b^	0.010	0.0315
Isoleucine	0.60 ± 0.05 ^a^	0.69 ± 0.02 ^b^	0.017	0.0344
Leucine	1.02 ± 0.08 ^a^	1.17 ± 0.03 ^b^	0.012	0.0315
Lysine	1.18 ± 0.11 ^a^	1.36 ± 0.03 ^b^	0.018	0.0344
Methionine	0.20 ± 0.11	0.19 ± 0.05	0.791	0.8743
Phenylalanine	0.52 ± 0.04 ^a^	0.60 ± 0.01 ^b^	0.011	0.0315
Threonine	0.60 ± 0.05 ^a^	0.70 ± 0.01 ^b^	0.011	0.0315
Valine	0.67 ± 0.05	0.75 ± 0.01	0.028	0.0406
NEAA				
Alanine	0.77 ± 0.07	0.89 ± 0.03	0.020	0.0350
Aspartic Acid	1.21 ± 0.10 ^a^	1.40 ± 0.03 ^b^	0.011	0.0315
Cysteine	0.15 ± 0.00	0.16 ± 0.01	0.210	0.2450
Glutamic Acid	2.02 ± 0.15 ^a^	2.33 ± 0.06 ^b^	0.007	0.0315
Glycine	0.64 ± 0.07	0.75 ± 0.08	0.045	0.0591
Proline	0.68 ± 0.03	0.74 ± 0.05	0.048	0.0593
Serine	0.55 ± 0.04 ^a^	0.63 ± 0.02 ^b^	0.009	0.0315
Tyrosine	0.36 ± 0.05	0.43 ± 0.03	0.029	0.0406
TAA	12.41 ± 1.08 ^a^	14.26 ± 0.37 ^b^	0.016	0.0344
EAA	6.04 ± 0.60	6.93 ± 0.20	0.027	0.0406
EAA/TAA, %	0.49 ± 0.01	0.49 ± 0.00	0.956	0.9560
EAA/NEAA, %	0.95 ± 0.02	0.95 ± 0.02	0.949	0.9560

Notes: ^(1)^ EAA, essential amino acids; NEAA, non-essential amino acids; TAA, total amino acids. Values with different superscript letters in the same row differ significantly (*p* < 0.02 and FDR < 0.05). Data are expressed as mean ± standard deviation. Each replicate was regarded as an experimental unit for statistical analysis (*n* = 5).

**Table 10:** Effects of different supplemental levels of EH-BSFLH on blood routine parameters of broilers.

Parameters ^(1)^	Dietary Treatments			*p*-Value	FDR
	Control	2% EH-BSFLH	6% EH-BSFLH		
WBC, ×10^9^/L	69.54 ± 6.00	66.80 ± 9.26	66.78 ± 4.07	0.687	0.9235
LYM#, ×10^9^/L	54.41 ± 3.61	52.17 ± 6.73	52.11 ± 2.42	0.580	0.9235
MID#, ×10^9^/L	4.02 ± 0.51	3.83 ± 0.67	3.75 ± 0.35	0.627	0.9235
GRA#, ×10^9^/L	11.07 ± 2.01	10.82 ± 2.35	10.91 ± 1.47	0.974	0.9740
LYM%, %	78.52 ± 1.81	78.69 ± 2.28	78.41 ± 1.55	0.962	0.9740
MID%, %	5.78 ± 0.30	5.63 ± 0.32	5.56 ± 0.18	0.341	0.8333
GRA%, %	15.75 ± 1.59	15.66 ± 2.05	16.03 ± 1.39	0.912	0.9740
RBC, ×10^12^/L	2.183 ± 0.116	2.125 ± 0.236	2.150 ± 0.019	0.785	0.9235
HGB, g/L	93.38 ± 3.26	93.19 ± 6.42	94.95 ± 1.66	0.702	0.9235
MCHC, g/L	325.57 ± 4.85	339.29 ± 24.23	336.43 ± 8.61	0.228	0.8333
MCH, pg	42.84 ± 1.28	44.17 ± 3.11	44.18 ± 0.85	0.375	0.8333
MCV, fL	131.51 ± 2.51	130.47 ± 3.45	131.41 ± 2.80	0.767	0.9235
RDW-CV, %	10.66 ± 1.40	11.44 ± 1.76	9.43 ± 1.25	0.063	0.6850
RDW-SD, fL	64.78 ± 11.47	68.29 ± 11.07	56.19 ± 10.58	0.137	0.6850
HCT, %	28.61 ± 1.06	27.70 ± 2.85	28.23 ± 0.52	0.633	0.9235
PLT, ×10^9^/L	42.71 ± 6.37	40.14 ± 5.07	36.00 ± 6.27	0.130	0.6850
MPV, fL	5.57 ± 0.14	5.60 ± 0.15	5.50 ± 0.16	0.426	0.8520
PDW, fL	10.17 ± 0.54	10.54 ± 0.62	10.24 ± 0.35	0.369	0.8333
PCT, %	0.024 ± 0.004	0.0228 ± 0.0032	0.019 ± 0.0036	0.134	0.6850
P-LCR, %	4.70 ± 1.50	4.41 ± 2.14	3.46 ± 1.06	0.350	0.8333

Notes: ^(1)^ WBC, White Blood Cell count; LYM#, Absolute Lymphocyte count; MID#, Absolute MID (mid-sized cell) count; GRA#, Absolute Granulocyte count; LYM%, Lymphocyte percentage; MID%, Mid-sized cell percentage; GRA%, Granulocyte percentage; RBC, Red Blood Cell count; HGB, Hemoglobin; MCHC, Mean Corpuscular Hemoglobin Concentration; MCH, Mean Corpuscular Hemoglobin; MCV, Mean Corpuscular Volume; RDW-CV, Red Cell Distribution Width-Coefficient of Variation; RDW-SD, Red Cell Distribution Width-Standard Deviation; HCT, Hematocrit; PLT, Platelet count; MPV, Mean Platelet Volume; PDW, Platelet Distribution Width; PCT, Plateletcrit; P-LCR, Platelet-Large Cell Ratio. Data are expressed as mean ± standard deviation. Each replicate was regarded as an experimental unit for statistical analysis (*n* = 7).

**Table 11:** Effects of different supplemental levels of EH-BSFLH on serum biochemical parameters of broilers.

Parameters ^(1)^	Dietary Treatments ^(2)^		*p*-Value	FDR
	Control	6% EH-BSFLH		
GLU, mmol/L	12.23 ± 0.37 ^b^	11.40 ± 0.43 ^a^	0.012	0.0360
ALB, g/L	13.06 ± 0.36 ^b^	11.84 ± 0.63 ^a^	0.008	0.0320
ALT, U/L	2.62 ± 0.43 ^b^	1.90 ± 0.26 ^a^	0.017	0.0408
CK, U/L	1989.94 ± 117.20	2048.56 ± 55.21	0.353	0.4236
TG, mmol/L	0.70 ± 0.17	0.50 ± 0.15	0.099	0.1515
AST, U/L	281.06 ± 13.00 ^b^	252.30 ± 12.33 ^a^	0.007	0.0320
T-BIL, umol/L	15.58 ± 1.70	13.62 ± 1.63	0.101	0.1515
UA, umol/L	264.42 ± 65.16	247.02 ± 50.16	0.650	0.6580
TP, g/L	28.66 ± 1.03	26.20 ± 2.04	0.053	0.1060
TCHO, mmol/L	4.07 ± 0.16 ^b^	3.49 ± 0.25 ^a^	0.004	0.0320
GLB, g/L	15.60 ± 0.72	14.36 ± 1.45	0.139	0.1853
A/G	0.84 ± 0.03	0.83 ± 0.04	0.658	0.6580

Notes: ^(1)^ GLU, glucose; ALB, albumin; ALT, alanine aminotransferase; CK, creatine kinase; TG, triglycerides; AST, aspartate aminotransferase; T-BIL, total bilirubin; UA, uric acid; TP, total protein; GLB, globulin. ^(2)^ EH-BSFLH2 represents the experimental diet supplemented with 6% EH-BSFLH (days 1–21). Values with different superscript letters in the same row differ significantly (*p* < 0.02 and FDR < 0.05). Data are expressed as mean ± standard deviation. Each replicate was used as an experimental unit for statistical analysis (*n* = 7).

**Table 12:** Effects of different supplemental levels of EH-BSFLH on intestinal indices of broilers.

Parameters	Dietary Treatments			*p*-Value	FDR
	Control	2% EH-BSFLH	6% EH-BSFLH		
Duodenum relative weight, g/kg	10.60 ± 1.58	10.39 ± 0.89	9.83 ± 1.02	0.588	0.5880
Jejunum relative weight, g/kg	15.89 ± 1.24	16.42 ± 1.52	14.01 ± 0.82	0.022	0.1320
Ileum relative weight, g/kg	10.93 ± 1.44	11.17 ± 1.39	9.47 ± 0.41	0.086	0.2580
Duodenum relative length, cm/kg	28.88 ± 2.34	27.79 ± 2.80	27.07 ± 2.02	0.510	0.5880
Jejunum relative length, cm/kg	72.47 ± 7.55	68.81 ± 3.84	68.01 ± 3.48	0.393	0.5880
Ileum relative length, cm/kg	70.91 ± 9.34	70.75 ± 5.59	66.03 ± 3.82	0.445	0.5880

Notes: Values with different superscript letters in the same row differ significantly (*p* < 0.02 and FDR < 0.05). Data are expressed as mean ± standard deviation. Each replicate was regarded as an experimental unit for statistical analysis (*n* = 7).

**Table 13:** Effects of different supplemental levels of EH-BSFLH on intestinal morphology of broilers.

Parameters ^(1)^	Dietary Treatments		*p*-Value	FDR
	Control	6% EH-BSFLH		
Duodenum				
VH, μm	1525.65 ± 212.34	1769.01 ± 85.74	0.102	0.1836
CD, μm	229.73 ± 22.86 ^b^	177.55 ± 12.85 ^a^	0.012	0.0360
VH/CD	6.66 ± 0.94 ^a^	10.02 ± 1.10 ^b^	0.004	0.0360
Jejunum				
VH, μm	1202.41 ± 268.69	1416.52 ± 209.64	0.182	0.2340
CD, μm	219.06 ± 29.09	179.60 ± 12.32	0.066	0.1485
VH/CD	5.51 ± 1.07 ^a^	8.13 ± 0.98 ^b^	0.011	0.0360
Ileum				
VH, μm	913.57 ± 34.64	889.11 ± 160.84	0.784	0.7840
CD, μm	212.30 ± 19.60	191.52 ± 13.71	0.139	0.2085
VH/CD	4.35 ± 0.61	4.67 ± 1.02	0.606	0.6817

Notes: ^(1)^ VH, Villus Height; CD, Crypt Depth; VH/CD, Villus Height/Crypt Depth. Values with different superscript letters in the same row differ significantly (*p* < 0.02 and FDR < 0.05). Data are expressed as mean ± standard deviation. Each replicate was regarded as an experimental unit for statistical analysis (*n* = 7).

**Table 14:** Effects of different supplemental levels of EH-BSFLH on economic returns (Chinese Yuan CNY) of broilers.

Parameters/Broiler	Dietary Treatments			*p*-Value	FDR
	Control	2% EH-BSFLH	6% EH-BSFLH		
Feed cost, CNY	3.30 ± 0.19	3.49 ± 0.16	3.60 ± 0.21	0.020	0.024
EH-BSFLH cost, CNY	0.00 ± 0.00 ^a^	0.10 ± 0.00 ^b^	0.33 ± 0.02 ^c^	<0.001	0
Running cost, CNY	0.75 ± 0.00	0.75 ± 0.00	0.75 ± 0.00	1	1.0000
Total cost, CNY	4.05 ± 0.19 ^a^	4.34 ± 0.16 ^b^	4.69 ± 0.23 ^c^	<0.001	0
Revenue ^(1)^, CNY	16.36 ± 1.10 ^a^	17.23 ± 0.86 ^a^	18.73 ± 0.89 ^b^	<0.001	0
Profit, CNY	12.32 ± 0.97 ^a^	12.90 ± 0.76 ^a^	14.05 ± 0.69 ^b^	0.003	0.0045

^(1)^ According to market price released by Ministry of Agriculture and Rural Affairs of the People’s Republic of China (https://xmsyj.moa.gov.cn/jcyj/202605/t20260526_6484455.htm (accessed on 27 September 2026)). Notes: Values with different superscript letters in the same row differ significantly (*p* < 0.02 and FDR < 0.05). Data are expressed as mean ± standard deviation. Each replicate was regarded as an experimental unit for statistical analysis (*n* = 7).

## Data Availability

The original contributions presented in this study are included in the article/Supplementary Materials. Further inquiries can be directed to the corresponding author.

## Supplementary Materials

The following supporting information can be downloaded at: https://www.mdpi.com/article/10.3390/vetsci13101030/s1, Table S1: Microbiological detection of EH-BSFLH; Table S2: Descriptive statistics and pairwise comparison *p*-values of nutritional composition and key nutrient indices for different dietary EH-BSFLH groups; Table S3: Body weight of broilers at sampling; Table S4: Descriptive statistics and pairwise comparison *p*-values of production performance different dietary EH-BSFLH groups; Table S5: Descriptive statistics and pairwise comparison *p*-values of slaughter performance different dietary EH-BSFLH groups; Table S6: Descriptive statistics and pairwise comparison *p*-values of meat quality different dietary EH-BSFLH groups; Table S7: Descriptive statistics and pairwise comparison *p*-values of blood routine parameters different dietary EH-BSFLH groups; Table S8: Descriptive statistics and pairwise comparison *p*-values of intestinal indices different dietary EH-BSFLH groups; Table S9: Descriptive statistics and pairwise comparison *p*-values of economic returns different dietary EH-BSFLH groups; Table S10: Analysis of economic returns different dietary EH-BSFLH groups.
